# Interventional radiology using endoscopy: Blood supply route-targeted endoscopic injection sclerotherapy with multiple ligations for giant esophagogastric varices

**DOI:** 10.1016/j.radcr.2023.03.037

**Published:** 2023-04-19

**Authors:** Fumio Chikamori, Koji Kojima, Kunihisa Uchita, Niranjan Sharma

**Affiliations:** a Department of Surgery, Japanese Red Cross Kochi Hospital, 1-4-63-11 Hadaminamimachi 780-8562, Kochi, Japan; b Department of Gastroenterology, Japanese Red Cross Kochi Hospital, 1-4-63-11 Hadaminamimachi 780-8562, Kochi, Japan; c Adv Train Gastroint & Organ Transp Surgery, 12 Scotland St Dunedin 9016, New Zealand

**Keywords:** Partial splenic embolization, Splanchnic caput Medusae, Esophagogastric varices, Endoscopic injection sclerotherapy with ligation, Portal hypertension, Intravariceal injection sclerotherapy

## Abstract

A case of high-risk giant esophagogastric varices was treated by blood supply route-targeted endoscopic injection sclerotherapy with multiple ligations (EISML). An endoscope was inserted in the left lower semi-lateral position under general anesthesia in the digital subtraction angiography room. The C-arm was rotated to obtain a frontal view for fluoroscopy. Before puncturing the esophageal varices, the balloon attached to the tip of the endoscope was inflated to block the variceal blood flow. At puncture, an intravascular injection was confirmed fluoroscopically, and a total of 18 m of 5% ethanolamine oleate with iopamidol was injected retrogradely at 5-minute intervals from the esophagogastric varices to the root of the left gastric vein, maintaining stagnation for 25 minutes. The variceal site of the injection was ligated immediately after the removal of the needle to prevent variceal bleeding. Multiple variceal ligations were added to stop the variceal blood flow. Contrast-enhanced CT 3 days after EISML showed the thrombus formation in esophagogastric varices and the left gastric vein. The blood supply route-targeted EISML can be a feasible procedure for giant esophagogastric varices.

## Introduction

Endoscopic injection sclerotherapy (EIS) is a simple term, but intravaricel injection is different from extravariceal injection. In addition, the procedure of injecting into the blood supply route of varices [1,2] is completely different from the procedure limited to local injection [3,4]. Historically, the former is widely adopted in Japan. Whereas, the latter is widely adopted in Europe and the United States of America (USA). In Europe and the USA, the position of EIS in the treatment of esophagogastric varices is declining [5]. The abandonment of EIS and the adoption of only endoscopic variceal ligation (EVL) in the endoscopic treatment of esophagogastric varices remains a problem. EVL has a high recurrence rate and is not only ineffective in treating giant esophagogastric varices, but also has a risk of fatal bleeding [6]. Hybrid procedure; combining partial splenic embolization (PSE) and EIS with ligation (EISL) for esophagogastric varices has been reported [7]. In 2020, a new concept *“splanchnic caput Medusae”* was proposed: the spleen is its face and the portal collateral pathways are its snake hairs [8]. In the new concept, EISL is considered as the treatment of Medusae's hair. Because EISL blocks variceal blood flow, thrombus formation of the variceal blood supply route begins shortly after EISL [9]. However, in the case of giant esophagogastric varices, it is difficult to completely block variceal blood flow by standard EISL procedure [10]. We report a case in which a modified EISL procedure involving multiple ligations aiming obliteration of both giant esophagogastric varices and their blood supply route namely, a blood supply route-targeted EIS with multiple ligations (EISML) was successfully executed.

## Case

A 53-year-old male with hematemesis was transferred to the emergency department of our hospital. He had a history of alcohol and hepatitis C virus (HCV) related cirrhosis with ascites and was treated with sofosbuvir / velpatasvir and diuretics 1 year ago.

On admission, his temperature was 36.7°C, blood pressure was 112/70 mmHg, and heart rate was 95/min. His height was 170 cm, body weight was 70 kg, and body mass index was 24.2 kg/m^2^. He did not have jaundice and his consciousness level was lucid. Laboratory studies revealed hemoglobin 7.5 g/dL (normal range, 13.5-17.4); total leukocyte count 5580 /µL (3500-8000 /µL); platelet count 13.2×10^4^ /µL (12.3-33.1×10^4^ /µL); total bilirubin 0.7 mg/dL (0.3-1.3 mg/dL); albumin 3.2 g/dL (3.8-5.0 g/dL); aspartate transaminase (AST) 23 U/L (10-32 U/L); alanine transaminase (ALT) 22 U/L (5-27 U/L); prothrombin time (PT) 62.0% (70%-130 %); Mac-2 binding protein glycosylated isomers (M2BPGi) 3.99 COI (2+) (<1.00); serum ammonia (NH3) 61 µg/dL (12-66 µg/dL). The retention rate of indocyanine green at 15 minutes (ICG15) was 19 % (<10 %). The Child-Pugh score was 7 and the class was B. Hepatitis B surface antigen was negative. HCV-RNA was undetectable.

**Fig. 1:**
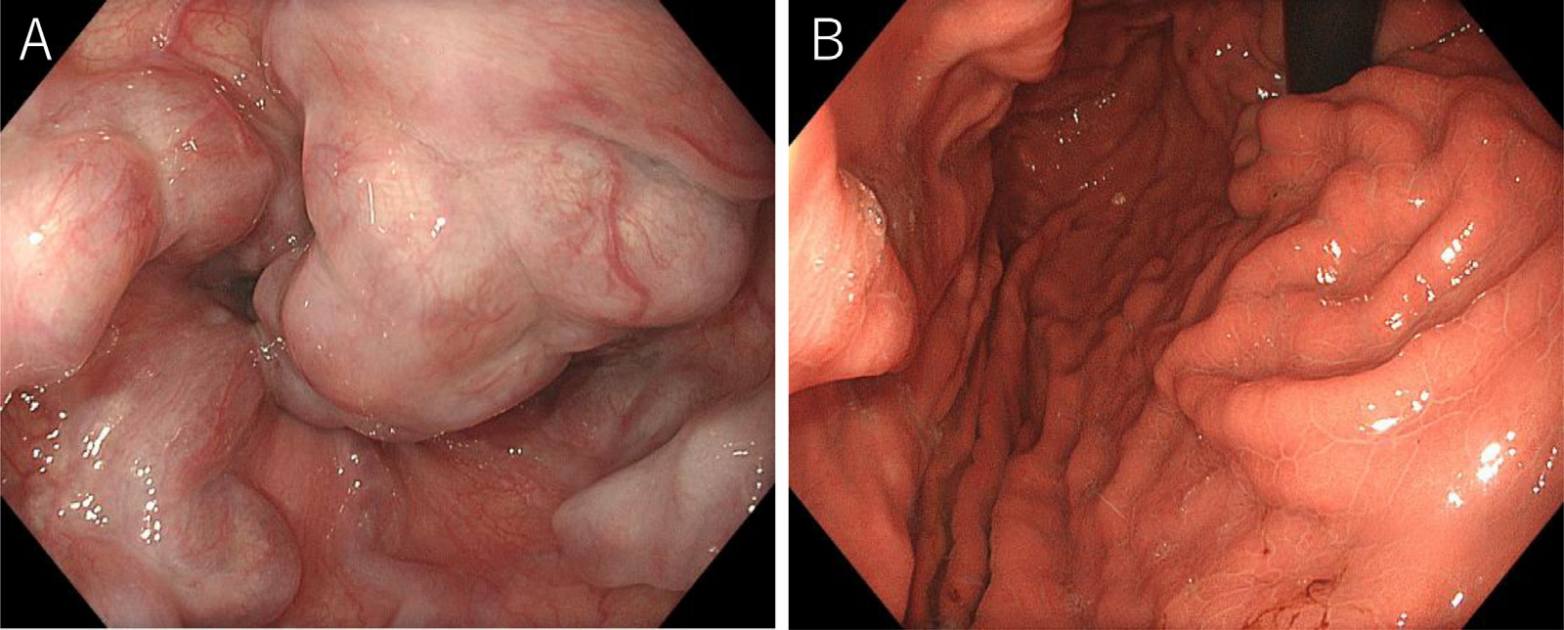
Endoscopic picture before stepwise PSE and EISML. (A) Endoscopic picture shows giant esophageal varices. (B) Endoscopic picture shows moderately - enlarged gastric varices at the cardia. EISML, endoscopic injection sclerotherapy with multiple ligations; PSE, partial splenic embolization.

**Fig. 2:**
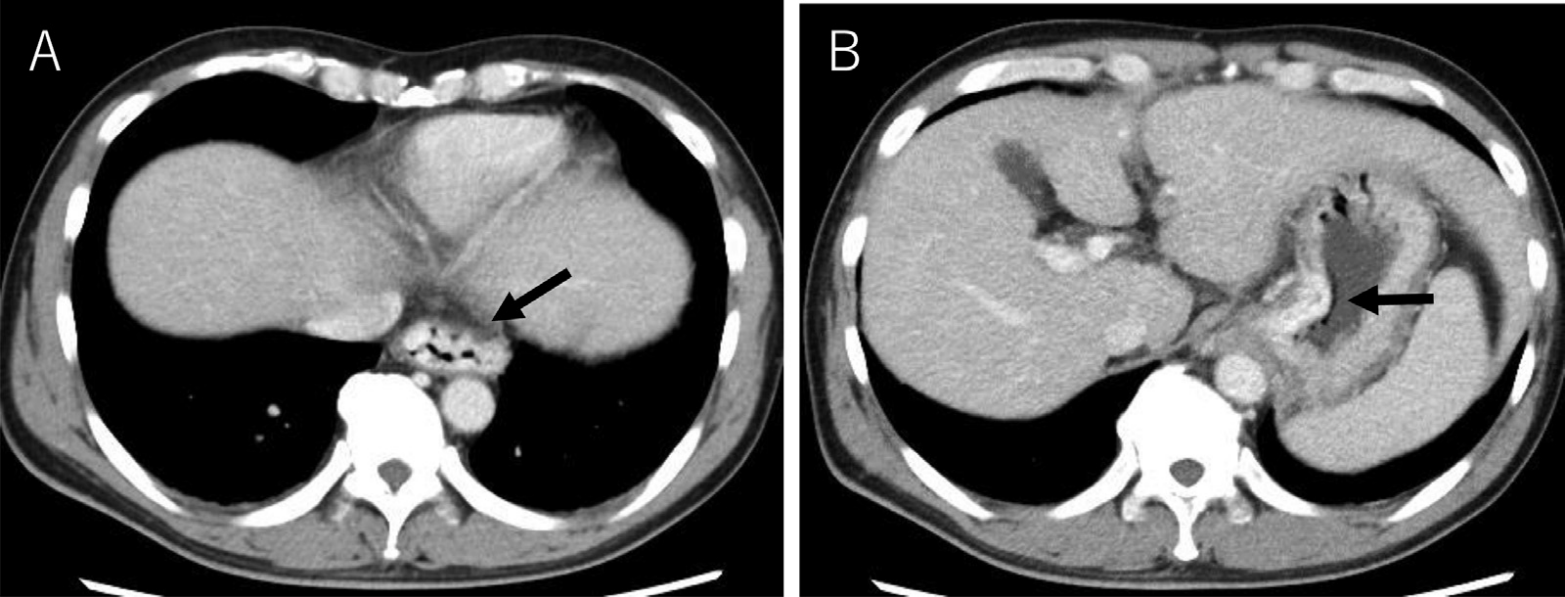
CE-CT image before stepwise PSE and EISML. (A) CE-CT image shows the giant esophageal varices (black arrow). (B) CE-CT image shows gastric varices at the cardia. EISML, endoscopic injection sclerotherapy with multiple ligations; PSE, partial splenic embolization.

**Fig. 3:**
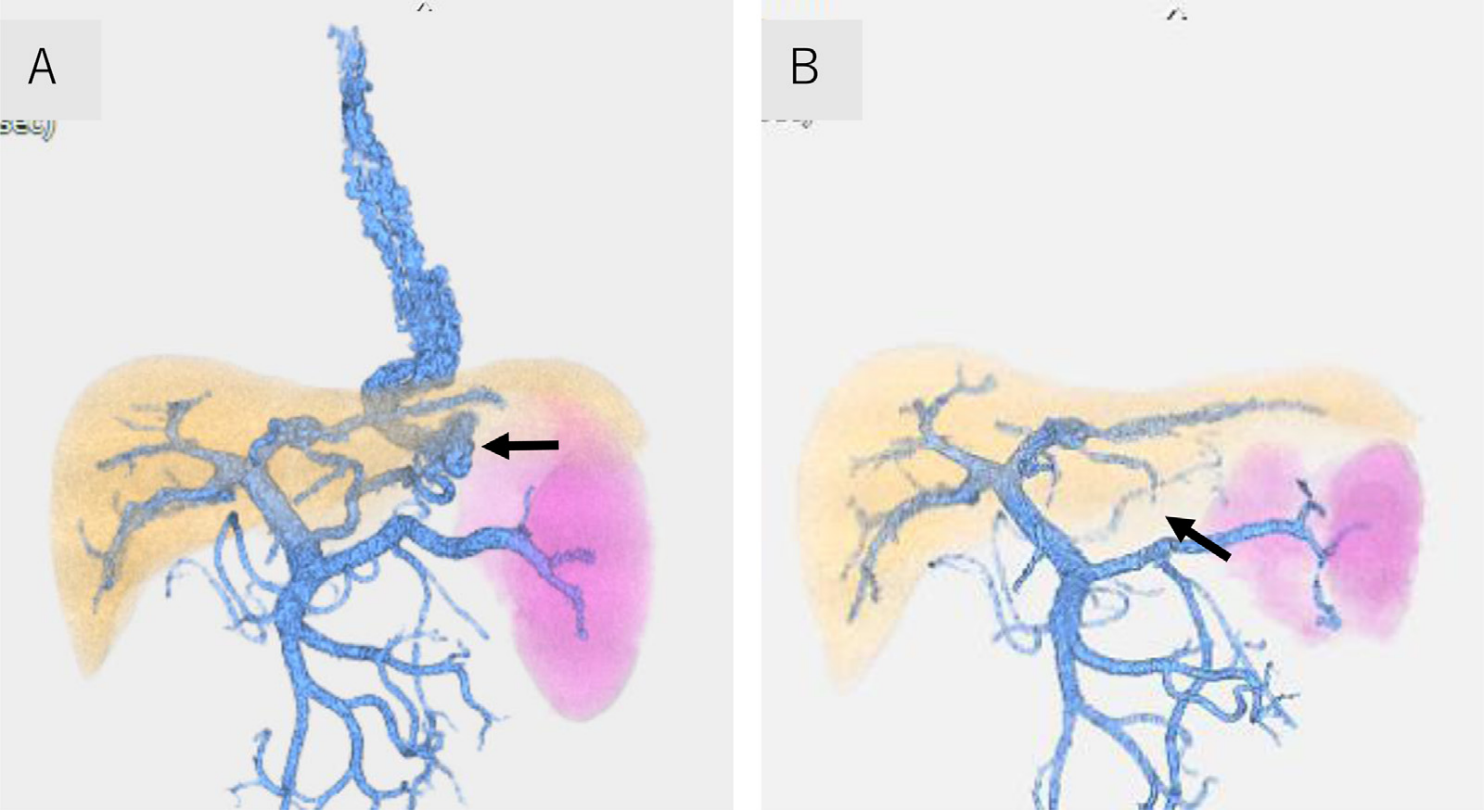
3D-CT reconstruction portal image before and after stepwise PSE and EISML. (A) 3D-CT reconstruction portal image before stepwise PSE and EISML shows the left gastric vein, gastric (arrow), and esophageal varices. (B) 3D-CT reconstruction portal image after stepwise PSE and EISML shows the disappearance of blood flow in esophagogastric varices. Partial thrombosis is observed in the left gastric vein (arrow). EISML, endoscopic injection sclerotherapy with multiple ligations; PSE, partial splenic embolization.

The patient's vital signs were stable after the initial IV infusion and blood transfusion. Emergency endoscopy confirmed giant esophagogastric varices with a fibrin plug, and hemostasis by EVL was performed. The gastric varices were located at the cardia (Figs. 1A and B). Abdominal contrast-enhanced CT (CE-CT) showed liver cirrhosis with giant esophagogastric varices (Figs. 2A and B). 3D-CT reconstruction portal image demonstrated that the esophagogastric varices were supplied by the left gastric vein. The spleen volume was 598 mL, and the liver volume was 1252 mL; giving a spleen/liver volume ratio of 0.5 (Fig. 3A).

As the patient had splenomegaly secondary to portal hypertension, stepwise PSE [8] followed by elective treatment of esophagogastric varices was planned. The venous phase of superior mesenteric arteriography demonstrated the hepatofugal left gastric vein and giant esophagogastric varices. By 2-step PSE, hepatic venous pressure gradient (HVPG) decreased from 20 to 10 mmHg and the spleen/liver volume ratio decreased from 0.5 to 0.2. However, there was no change in the morphology of esophagogastric varices, that remained giant and risky.

**Fig. 4:**
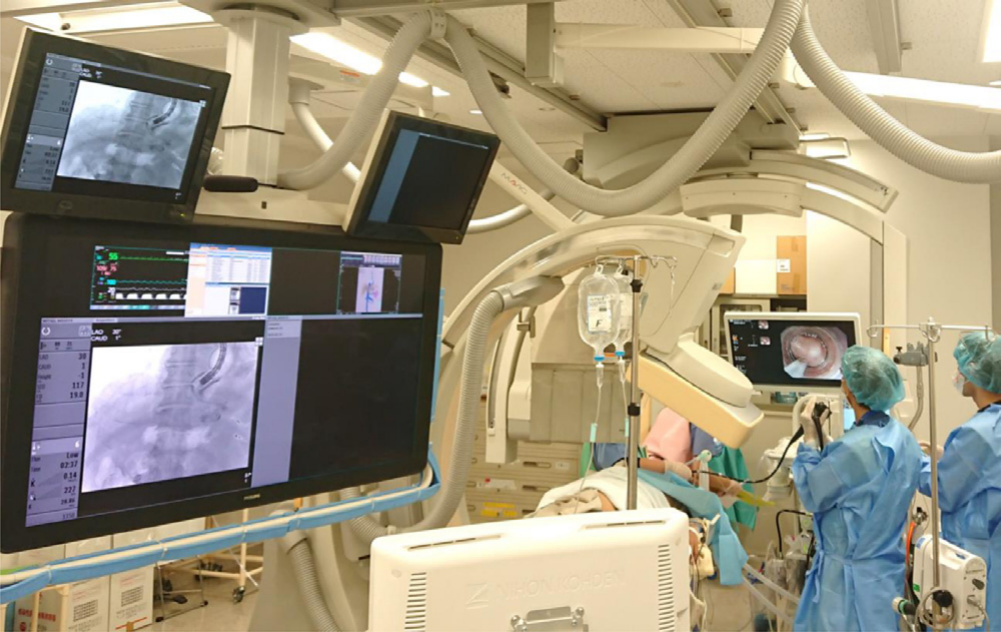
Posture and C-arm position during EISML. EISML, endoscopic injection sclerotherapy with multiple ligations.

**Fig. 5:**
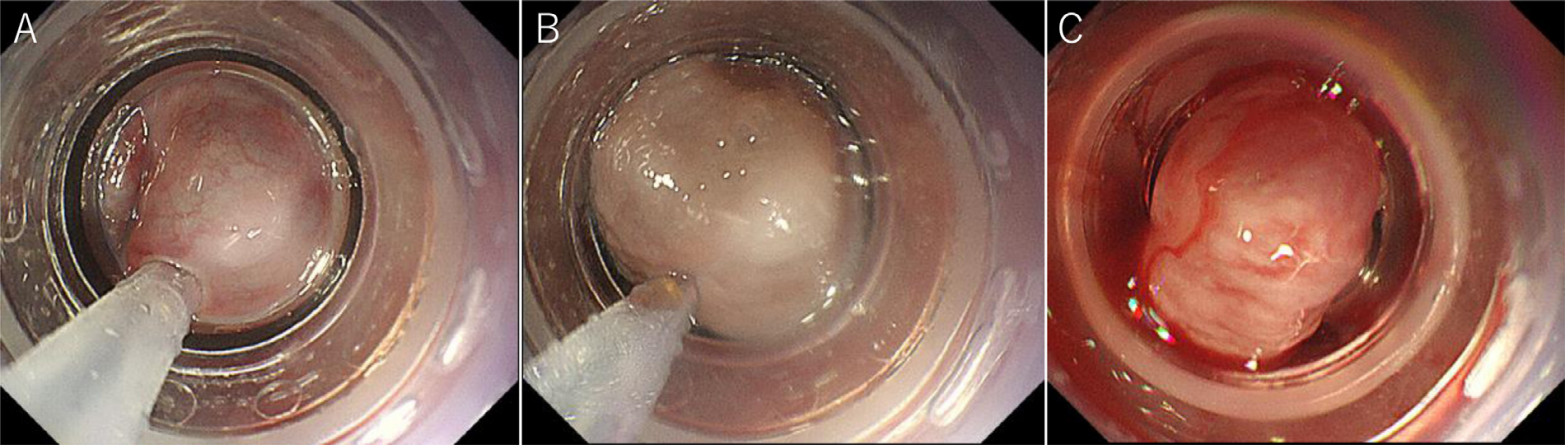
Endoscopic picture during EISML. (A) Endoscopic picture shows esophageal varices 3 minutes after needle puncture. (B) Endoscopic picture shows a stable needle in the esophageal varices 21 minutes after the puncture. (C) Endoscopic picture shows the variceal site of the puncture that was ligated immediately after the removal of the needle. EISML, endoscopic injection sclerotherapy with multiple ligations.

**Fig. 6:**
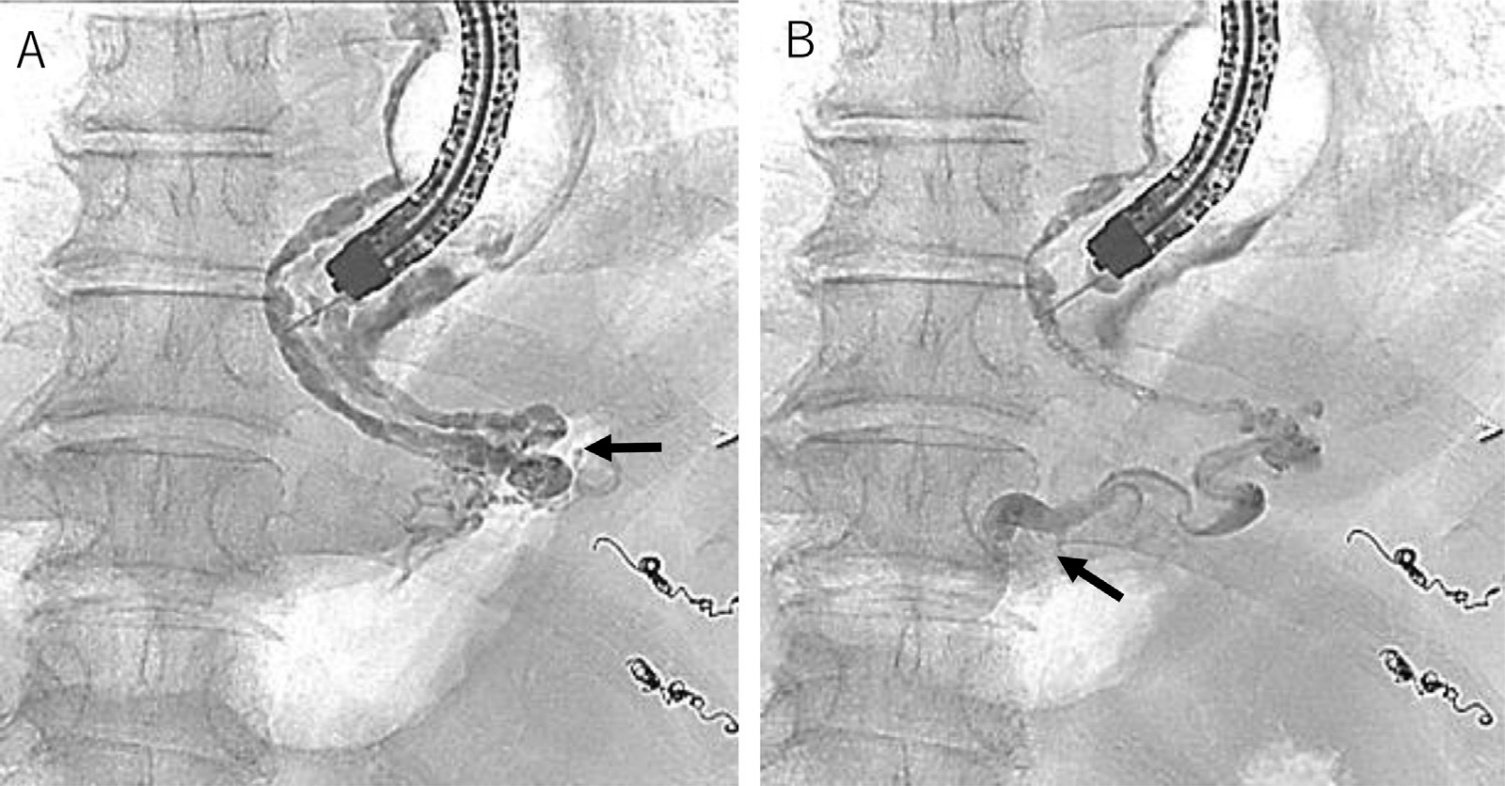
Retrograde endoscopic varicogram during EISML. (A) Retrograde endoscopic varicogram 3 minutes after needle puncture shows esophageal and gastric varices (arrow). Although there is only 1 injection site, all esophageal varices caudal to the balloon are filled with 5% EOI. (B) Retrograde endoscopic varicogram 21 minutes after needle puncture shows the left gastric vein (arrow). EISML, endoscopic injection sclerotherapy with multiple ligations.

Therefore, a blood supply route-targeted EISML under general anesthesia in the digital subtraction angiography room was performed. The patient lying in the left lower semi-lateral recumbent position, the endoscope was inserted followed by an over-tube insertion. The C-arm was rotated to obtain a front view for fluoroscopy (Fig. 4). Before puncturing the esophageal varices, the balloon attached to the tip of the endoscope was inflated to block the variceal blood flow. In the first puncture, backflow of blood was confirmed, and 2 mL of 5% ethanolamine oleate with iopamidol (5% EOI) was injected. However, it was determined to be extravascular by fluoroscopy, so the needle was removed and band ligation was applied to the same site to prevent bleeding. In the second puncture (Fig. 5A), an intravariceal injection was confirmed fluoroscopically, and a total of 18 mL of 5% EOI was retrogradely injected at 5-minute intervals from the esophagogastric varices to the root of the left gastric vein, and stagnation of the sclerosing agent was maintained for 25 minutes (Figs. 6A and B). The variceal site of the injection was ligated immediately after the removal of the needle to protect against variceal bleeding (Figs. 5B and C). Fourteen more variceal ligations were added to stop the variceal blood flow.

Then, the patient was placed in the supine position and hepatic venous catheterization via the right antecubital vein was performed. Since HVPG after EISML was 12 mmHg, a third PSE was added to lower it to 10 mmHg. The venous phase of superior mesenteric arteriography just after EISML demonstrated that the left gastric vein and esophagogastric varices were not visualized.

**Fig. 7:**
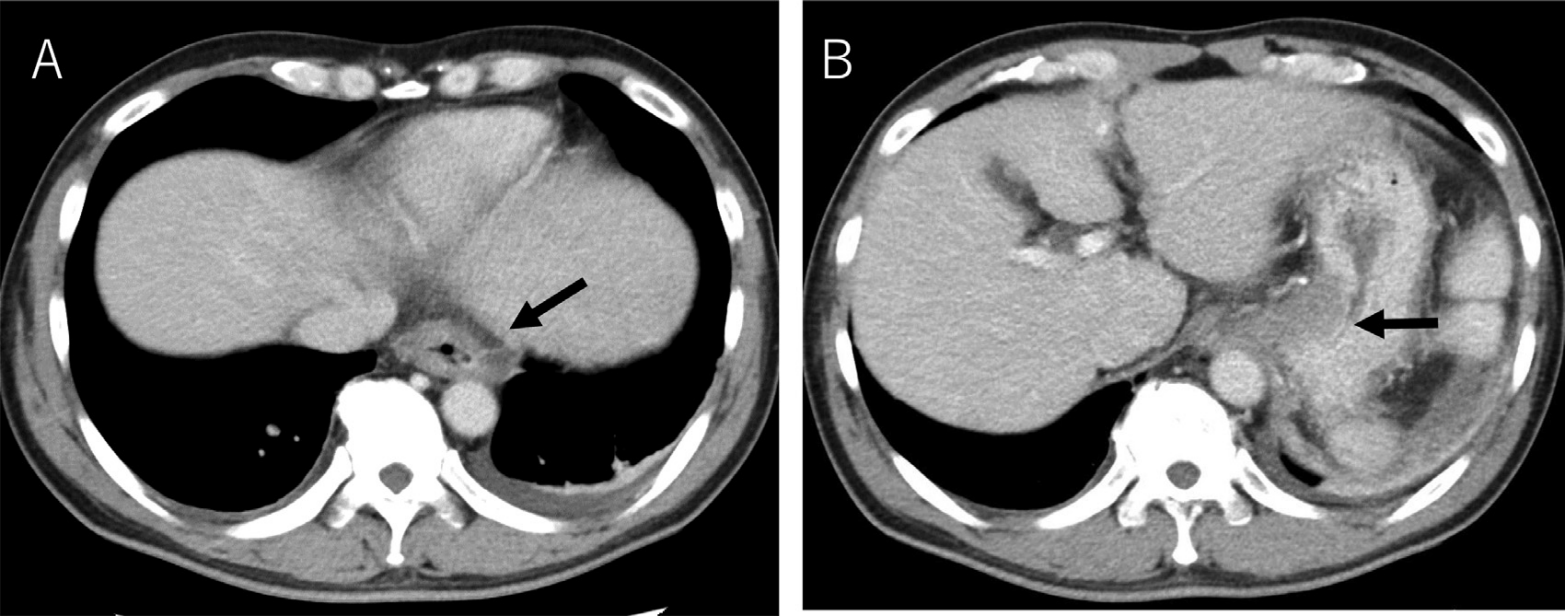
CE-CT image after stepwise PSE and EISML. (A) CE-CT image shows esophageal variceal thrombus formation (black arrow). (B) CE-CT image shows gastric variceal thrombus formation at the cardia. EISML, endoscopic injection sclerotherapy with multiple ligations; PSE, partial splenic embolization.

**Fig. 8:**
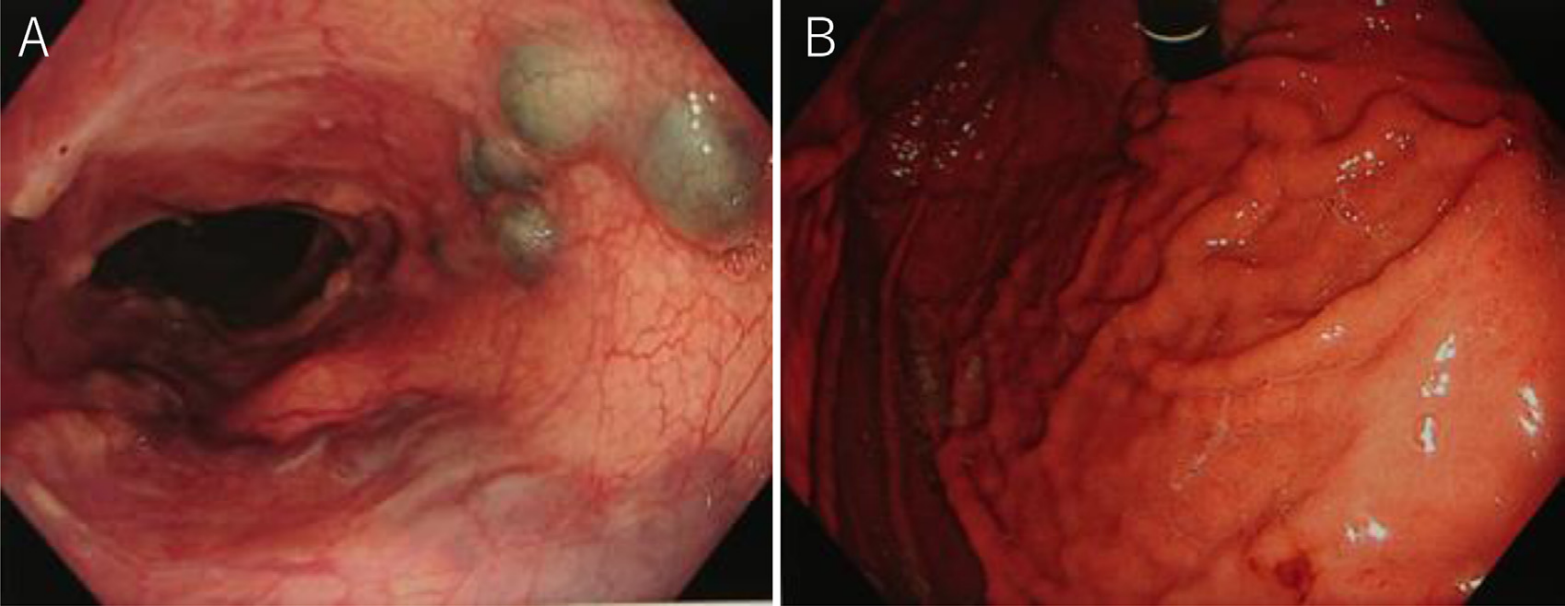
Endoscopic picture 2 weeks after EISML. (A) Endoscopic picture shows thrombi and marked reduction of esophageal varices. (B) Endoscopic picture shows the reduction of gastric varices. EISML, endoscopic injection sclerotherapy with multiple ligations.

CE-CT 3 days after EISML showed the thrombus formation in esophagogastric varices and the left gastric vein (Figs. 7A and B). 3D-CT reconstruction portal image revealed that the portal system reversed to almost normal form (Fig. 2B). The spleen volume was 286 mL, and the liver volume was 1354 mL; giving a spleen/liver volume ratio of 0.2. The patient's postoperative course was uneventful and was discharged 8 days after EISML. Endoscopy 2 weeks after EISML confirmed thrombi and marked reduction of esophagogastric varices (Figs. 8A and B).

## Discussion

A case of high-risk giant esophagogastric varices was treated by a blood supply route-targeted EISML. The concept of the procedure is interventional radiology (IVR) using endoscopy.

Is there a fully established therapeutic strategy for giant esophagogastric varices? The answer is no. In Europe and the USA, the position of EIS in the treatment of esophagogastric varices has declined significantly. The reason is that EVL was superior to EIS 4). However, these results may be due to the fact that EIS procedures in Europe and the USA were limited to local injection [[3], [4], [11]]. In Japan, EIS procedures to obliterate both varices and their blood supply routes have been performed for a long time with good results [12]. The amount of sclerosant injected has been controlled by retrograde varicography using a balloon attached to the tip of the endoscope [1,2]. The procedure popular in Japan might be better renamed as the blood supply route-targeted EIS. Its procedural concept is IVR using endoscopy. However, it has not become popular in Europe and the USA, because it requires skill, fluoroscopy, and knowledge of portal collateral anatomy.

According to the “splanchnic caput Medusae” concept [8], the spleen is considered to be its face; and the portal collateral pathways are its snake hairs.

Esophagogastric varices and the left gastric vein are the snake-hair unit of transformation and should be treated rationally. Retrograde endoscopic varicography [1,2] is useful to identify the blood supply routes into which 5%EOI is injected. The optimal amount of 5%EOI can be determined by knowing where and how much 5%EOI is injected and how long it has been stagnant. Retrogradely injected 5% EOI destroys vascular endothelial cells 30 seconds after injection into the vessels, and a few hours later red blood cells and fibrin accumulate in the damaged vessels [13]. With blood supply route-targeted EIS alone, variceal blood flow reopens immediately after a 5% EOI injection. It takes time for the thrombus to obliterate the varices and their blood supply, and complete obliteration may not be achieved. Thus, even with the blood supply route-targeted EIS, it is difficult to treat giant esophagogastric varices in a single treatment session due to their high blood flow. On the other hand, continuous blockage of variceal blood flow with EISML contributes to accelerated thrombus formation.

Retrograde transvenous obliteration is not indicated for gastric varices continuing to the esophageal varices due to difficulty of access. Historically, retrograde transvenous obliteration for isolated gastric varices has been based on the concept of the blood supply route-targeted EIS [14]. Conversely, if the concept of retrograde transvenous obliteration is accepted in Europe and the USA, the concept of EISML will also be accepted. For transvenous retrograde gastric variceal obliteration, the use of coils has been attempted to block blood flow and form a complete thrombus in a short period of time [15]. This concept is the same as EISML for giant esophagogastric varices.

In the case of esophagogastric varices that are refractory to endoscopic treatment, embolization of the blood supply routes by percutaneous transhepatic (PTO) or transileocolic vein obliteration (TIO) is sometimes required [16]. The blood supply route-targeted EISML has the advantage of simultaneous obliteration of this supply route. In PTO and TIO, the obliteration of the blood supply side is dominant, while in EISML, the obliteration of the variceal side is dominant as shown in Figures 6A and B. In this case, the gastric varices at the cardia were continuous with the esophageal varices, so they could be treated at the same time. The purpose of this procedure is to treat the esophagogastric varices and their blood supply with a single needle puncture.

Rebleeding can be prevented by setting the HVPG to 12 mmHg or less [17]. Transjugular intrahepatic portosystemic shunt (TIPS) is a symptomatic therapy to reduce portal venous pressure in bleeding esophageal varices and has the potential to induce hyperammonemia and encephalopathy as side effects. From the perspective of the splanchnic caput Medusae concept, TIPS is an addition of the snake hair unit, which is contrary to the normal form. Therefore, TIPS should not be easily applied [5]. PSE is considered the treatment of the face of Medusae. PSE not only increases platelet count but also reduces the spleen/liver volume ratio, portal venous pressure, and splenic venous blood flow volume [18]. The advantages of the hybrid procedure combining endoscopic treatment and PSE for esophagogastric varices have been reported [8]. This case represents an elective blood supply route-targeted EISML in combination with a stepwise PSE.

Because confirmation of morphologic changes by endoscopy takes time, CE-CT and 3D-CT reconstruction images are recommended for the evaluation of the blood supply route-targeted EISML. The blood supply route-targeted EISML can be a feasible procedure for giant esophagogastric varices.

## Patient consent

Written informed consent was obtained from the patient for publication of this case report and accompanying images.
